# Tertiary Lymphoid Structure-Associated B Cells are Key Players in Anti-Tumor Immunity

**DOI:** 10.3389/fimmu.2015.00067

**Published:** 2015-02-23

**Authors:** Claire Germain, Sacha Gnjatic, Marie-Caroline Dieu-Nosjean

**Affiliations:** ^1^Laboratory Cancer, Immune Control and Escape, Cordeliers Research Center, INSERM UMRS1138, Paris, France; ^2^UMRS1138, University Pierre and Marie Curie, Paris, France; ^3^UMRS1138, University Paris Descartes, Paris, France; ^4^Division of Hematology, Oncology and Immunology, The Tisch Cancer Institute, Icahn School of Medicine at Mount Sinai, New York, NY, USA

**Keywords:** tertiary lymphoid structure, cancer, B cell, antibody, dendritic cell, anti-tumor immunity, biomarker, immunotherapy

## Abstract

It is now admitted that the immune system plays a major role in tumor control. Besides the existence of tumor-specific T cells and B cells, many studies have demonstrated that high numbers of tumor-infiltrating lymphocytes are associated with good clinical outcome. In addition, not only the density but also the organization of tumor-infiltrating immune cells has been shown to determine patient survival. Indeed, more and more studies describe the development within the tumor microenvironment of tertiary lymphoid structures (TLS), whose presence has a positive impact on tumor prognosis. TLS are transient ectopic lymphoid aggregates displaying the same organization and functionality as canonical secondary lymphoid organs, with T-cell-rich and B-cell-rich areas that are sites for the differentiation of effector and memory T cells and B cells. However, factors favoring the emergence of such structures within tumors still need to be fully characterized. In this review, we survey the state of the art of what is known about the general organization, induction, and functionality of TLS during chronic inflammation, and more especially in cancer, with a particular focus on the B-cell compartment. We detail the role played by TLS B cells in anti-tumor immunity, both as antigen-presenting cells and tumor antigen-specific antibody-secreting cells, and raise the question of the capacity of chemotherapeutic and immunotherapeutic agents to induce the development of TLS within tumors. Finally, we explore how to take advantage of our knowledge on TLS B cells to develop new therapeutic tools.

## Introduction

Tumors represent a complex microenvironment that not only includes tumor cells but also stromal cells, blood vessels, and different immune cell populations, reflecting the capacity of the immune system to sense tumor cells (1). The major role played by the immune system in tumor surveillance and control has been extensively documented. Since the discovery of T cells, B cells, and antibodies specific for tumor antigens (2–6), several clinical studies have clearly demonstrated that a high density of T-cell or B-cell subsets within the tumor microenvironment is associated with increased patient survival (1, 7, 8), such as in colorectal cancer (9), primary cutaneous melanoma (10), breast cancer (11–14), non-small cell lung cancer (NSCLC) (15, 16), head and neck cancer (17), and ovarian cancer (18–20).

Regarding the development of adaptive anti-tumor immune responses (but it is also true for any other kind of disorders), the dogma was that infiltrating anti-tumor immune cells are educated to recognize tumor antigens and proliferate at distance of the tumor site, in conventional secondary lymphoid organs (SLOs), and are then recruited to the tumor microenvironment to exert their anti-tumor activity. SLOs are highly specialized tissues that arise at predetermined locations during embryogenesis and persist all over the life. They include lymph nodes (LNs), spleen and mucosal-associated lymphoid tissues (MALTs), namely Peyer’s patches, tonsils, and nasal-associated lymphoid tissues (NALTs) or bronchus-associated lymphoid tissues (BALTs). However, it is now established that immune responses can also take place at distance of SLOs, in tertiary lymphoid structures (TLS), also called tertiary lymphoid organs (TLOs) or, in the case of the lung, (T)i-BALTs, for (tumor-) induced BALTs (21–23). As opposed to SLOs, TLS are defined as transient ectopic lymphoid organizations that can develop after birth in non-lymphoid tissues, in situations of chronic inflammation. This phenomenon is usually termed lymphoid “neogenesis” by opposition to lymphoid organogenesis, which refers to SLO development (24, 25). TLS have already been observed in several infectious diseases, graft rejection, autoimmune disorders [including rheumatoid arthritis (RA) and multiple sclerosis], allergy, and cancer (25–27). They have also been described in different lung inflammatory diseases, such as fibrosis, pneumonia, hypersensitivity pneumonitis, diffuse pan-bronchiolitis, and tobacco-induced inflammation (28), and in the adventitia of human atherosclerotic arteries (29–31). It has been proposed that the presence of TLS can be observed in every situation when the inflammatory response initiated is unable to eradicate the associated antigen (32). This inability might be the result of an efficient escape of a pathogen antigen to immune surveillance, and/or of a permanent replenishment of a self or tumor antigen by the tissue. The difficulty in eradicating antigens would lead to a chronic inflammation, characterized by a sustained infiltration of immune cells, and to the development of TLS as sites for the generation of a local more efficient immune response. Because these structures turn off after the resolution of the inflammation, they have been proposed as a genuine inflammation marker (27). In autoimmune diseases and transplantation, the presence of TLS is respectively associated with disease exacerbation, poor outcome (33), chronic rejection, and anti-graft responses (34–37). On the opposite, T- and B-cell responses initiated within TLS during infections are associated with pathogen clearance, increased survival, and reduced morbidity (21). Finally, an increasing number of studies demonstrated over the past decades that the presence of TLS in the tumor microenvironment is associated with anti-tumor immune responses and prolonged patient survival (22, 27, 38–44). Yet, the events that influence immune cell infiltration and TLS formation are still incompletely understood.

In this review, we discuss the factors favoring the recruitment of immune cells and their organization in TLS by comparison with what is known about conventional SLOs. We detail the role played by TLS B cells in anti-tumor immunity, both as tumor antigen-specific antibody-secreting cells and antigen-presenting cells (APCs). Finally, we raise the question of the capacity of chemotherapeutic agents and/or new immunotherapeutic drugs to induce the development of TLS within tumors, and how to take advantage of what we know now from these structures to develop new therapeutic tools.

## TLS Organization and Formation: How Far a TLS is Like a LN?

### TLS display the same organization as conventional SLOs

#### LN, a prototypical SLO

Lymph nodes are encapsulated structures mainly composed of T cells and B cells, dendritic cells (DCs), a network of fibroblastic reticular cells (FRCs) and fibers (45), and two specialized lymphatic and vascular systems: lymphatic vessels and high endothelial venules (HEVs), the latter specifically allowing the recruitment of naive T and B lymphocytes, but also plasmacytoid DCs (pDCs) and the precursors of conventional (CD11c^hi^) DCs (pre-cDCs) into the LN [reviewed in Ref. (46)].

Within LNs, B, and T cells are segregated into distinct functional areas: a B-cell area mainly composed of densely packed B cells and follicular DCs (FDCs) forming follicles, and a T-cell area mainly composed of less dense accumulation of T cells and DCs (25). While DCs prime T cells through the presentation of antigen-derived peptides within major histocompatibility complex (MHC) class I and II molecules, FDCs shape the B-cell response by presenting unprocessed antigens in the form of antigen-antibody complexes that they trap through their complement or fragment constant receptors (FcRs). After antigen encounter, activated B cells organize into a germinal center (GC), which is a site of active B-cell proliferation, class switch recombination (CSR), and somatic hyper-mutation (SHM), allowing the generation of B-cell clones highly specific for the antigen. A particular subset of T helper (Th) cells, namely T follicular helper (Tfh) cells, plays also a major role in GC responses, by delivering key signals for GC B-cell survival and differentiation [reviewed in Ref. (47)].

#### TLS, similarities and differences with LNs

**Figure 1:**
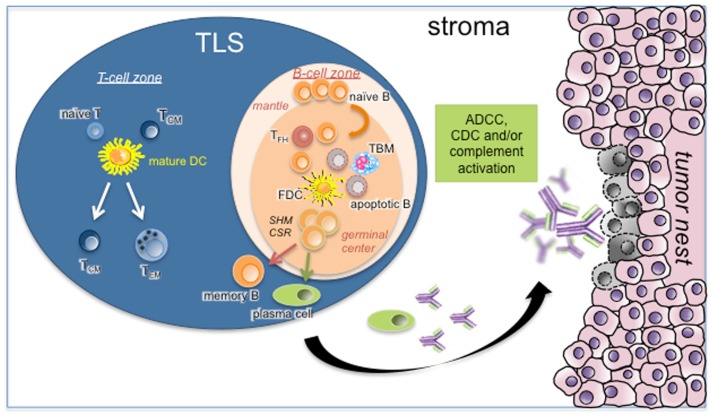
**Role of TLS B cells in the initiation of a protective anti-tumor immune response**. As in canonical SLOs, TLS may represent a critical site where specific T and B cells can undergo terminal differentiation into effector cells in the T- and B-cell-rich areas, respectively. In the latter case, germinal center B cells establish intimate interactions with FDCs and Tfh in the course of differentiation into memory B cells and PCs. This step is under the activation of CSR and SHM machineries. Apoptotic B cells will be phagocytosed by TBM whereas fully differentiated B cells will leave TLS B-cell follicle to the tumor stroma. After binding to their cognate tumor antigens, antibodies will elicit ADCC, CDC, and/or complement activation. Abbreviations: ADCC, antibody-dependent cellular cytotoxicity; CDC, complement-dependent cytotoxicity; CSR, class switch recombination; FDC, follicular dendritic cell; SHM, somatic hyper-mutation; TBM, tingible-body macrophage; T_CM_, central-memory T cell; T_EM_, effector-memory T cell; T_fh_, follicular helper T cell; TLS, tertiary lymphoid structure.

Tertiary lymphoid structures were shown to display an overall organization very similar to that observed in SLOs. In NSCLC for example, we showed that TLS were composed of a T-cell-rich area, characterized by T cells forming clusters with mature DCs expressing the maturation marker DC-Lamp, and CD20^+^ B cells, organized in B-cell follicle (Figure 1) (22, 39). Like in conventional LNs, B-cell follicles were composed of a mantle of IgD^+^ naive B cells, surrounding a GC identified by highly proliferating Ki67^+^ B cells and a network of CD21^+^ FDCs. Expression of activation-induced cytidine deaminase (AID) and of the transcription repressor Bcl6 by GC B cells, associated with CSR and SHM activities, was reported in several studies (32, 39, 40, 48). Finally, the presence of CD68^+^ tingible-body macrophages (specialized in the clearance of apoptotic B cells) and CD3^+^ CD4^+^ CXCL13^+^ Tfh cells could also be detected within TLS B-cell follicles (39, 43), as well as that of differentiated plasma cells (PCs) (39, 49, 50).

Like in SLOs, the presence of TLS is frequently associated with that of HEVs. HEVs can be easily distinguished from other types of blood vessels, thanks to a cuboidal endothelial cell lining and a set of molecules expressed by endothelial cells: peripheral node addressins (PNAd), which interact with L-selectin (CD62L) on lymphocytes and allow lymphocyte tethering to HEV endothelial cells and rolling along the HEV lumen; chemokines, including CCL19, CCL21, CXCL10, CXCL12, and CXCL13, known to determine the trafficking of lymphocytes but also their segregation into distinct areas within the LN (detailed below); and integrins and immunoglobulin superfamily adhesion molecules, which support the binding and migration of lymphocytes across HEVs (51, 52). Because HEVs are crucial for the recruitment of lymphocytes into LNs, they are also supposed to play a major role in tumor infiltration by immune cells and TLS formation (40, 53–56). Indeed, we observed in lung cancer that most TLS T cells and B cells (except GC B cells) expressed the PNAd ligand CD62L (53). Similarly, Martinet *et al*. demonstrated that the presence of HEVs in melanoma tumors was associated with high levels of lymphocyte infiltration (54). They notably showed that lymphocytes specifically infiltrated HEV-rich areas of melanoma tumors, and that the density of HEVs was strongly correlated with DC-Lamp^+^ mature DC density and CD3^+^, CD8^+^, and CD20^+^ tumor-infiltrating lymphocyte densities (55).

Interestingly, a major difference between SLOs and TLS is the absence of natural killer (NK) cells within TLS, as demonstrated in NSCLC using the NKp46 marker (57). To date, the presence of NK cells has never been reported in TLS regardless of the pathology concerned (cancer, autoimmunity, transplantation, or infectious diseases). In LNs, NK cells colocalize with DCs in the T-cell area (58), and form clusters with DCs and T cells getting primed shortly after infection, during the early phases of the adaptive immune response (59–61). On one hand, activated DCs can enhance NK cell proliferation, IFNγ secretion, and cytotoxic functions through the production of cytokines such as IL-15, IL-12, IL-18, and IFNα/β. On the other end, NK cells can either promote DC maturation through the production of IFNγ and TNFα, or kill immature DCs [reviewed in Ref. (62)]. Similarly, NK cells are thought to provide an early source of IFNγ that is needed to polarize the T-cell response (63), while newly primed Ag-specific CD4^+^ T cells can directly activate NK cells through the secretion of IL-2 (60, 64). These interactions are supposed to play a crucial role in shaping both innate and adaptive immune responses in LNs (65). The fact that intra-tumoral NK cells are not observed within cancer-associated TLS (57, 66) indicates that tumor-infiltrating NK cells may not influence T-cell response, and are not the cellular source of IFNγ detected within TLS.

### TLS neogenesis, what can we learn from LN organogenesis?

#### LN development

The development of LNs during embryogenesis depends on interactions between a unique subset of CD45^+^CD4^+^CD3^-^ hematopoietic cells, named lymphoid tissue-inducer (LTi) cells and resident stromal cells, qualified as lymphoid tissue-organizer (LTo) cells (67). In mice, van de Pavert *et al*. showed that the initial interaction between these two cell subsets was triggered by CXCL13 produced by stromal LTo cells at sites of lymphoid tissue formation, inducible upon retinoic acid and neuronal stimulation, and able to attract CXCR5-expressing LTi cells (68). The engagement of lymphotoxin (LT), a member of the TNF family expressed as an LTα1β2 heterotrimer on LTi cells, with lymphotoxin β receptor (LTβR) on stromal cells, then leads to the activation of two different pathways of the NF-κB transcription factor, resulting in the expression of high levels of the intercellular and vascular cell adhesion molecules ICAM-1 and VCAM-1, production of lymphoid chemokines (CCL19, CCL21, and CXCL13) and cytokines, and the development of both HEVs and lymphatic vessels (51, 69–72). The local expression of lymphoid chemokines by stromal LTo cells allows the subsequent attraction and trafficking of B, T, and DCs to the LN (67, 68). These three homeostatic chemokines not only govern the recruitment but also the positioning of immune cells within the LN. While CCL19 and CCL21 are expressed by the T-cell-zone stromal cells, and interact both with CCR7 to orchestrate the homing of naive and central-memory T cells and DCs to the T-cell compartment, CXCL13 is expressed by the B-cell-zone stromal cells, and allows the recruitment of CXCR5^+^ B cells and Tfh to the follicular compartment (73). Of note, CXCL13 was also shown to promote LTα1β2 expression on the surface of LTi cells, which amplifies the LTβR signaling in stromal LTo cells, resulting in a positive and stable feedback loop (67). Another feedback loop involving CXCL13 and LTα1β2 was also observed between FDCs and B cells, which are themselves strong producers of LTα and LTβ (74). LTα1β2 expression by B cells plays notably a major role in the establishment of the FDC network and in B-cell follicle organization. However, it has been demonstrated that membrane-bound LTα1β2 on B cells was a key regulator of CXCL13 production by FDCs, which in turn induces an upregulation of LTα1β2 on B cells (75).

HEV endothelial cells also express LTβR, and several studies have demonstrated that continuous engagement of LTβR on HEVs by LTα1β2^+^ cells is critical for the induction and maintenance of HEV gene expression and HEV endothelial cell morphology (70, 76). In mice, B cells (71, 77) and CD11c^+^ DCs (78) were both demonstrated as major sources of LTα1β2 for HEV regulation in lymphoid tissues. CD11c^+^ DCs in particular were shown to play an essential role in lymphocyte homing to the LNs, as a direct or indirect source of pro-angiogenic factors favoring the development of HEVs (79, 80). Webster *et al*. notably showed in immunized mice that CD11c^+^ DCs were able to induce increased vascular endothelial growth factor (VEGF) levels in LNs, favoring endothelial cell proliferation including HEV endothelial cell growth, associated with increased immune cell entry (81). In a further study, the same group showed that CD11c^+^ DC-induced vascular growth in this model is at least in part mediated by the production of IL-1β by CD11c^+^ DCs that can directly stimulate FRC proliferation and VEGF production (82). Similarly, activated B cells are able to secrete the pro-lymphangiogenic factor VEGF-A, and to drive lymphangiogenesis in inflamed LNs (83).

#### TLS neogenesis

The physiological events that lead to TLS formation are still unclear, in particular in cancer. Numerous studies in inflammatory disorders tried to determine if the same cell types and/or molecules involved in lymphoid organogenesis, could similarly be implicated in TLS neogenesis. If the presence of the *bona fide* LTi cells does not seem to be required for TLS induction in CCL21-transgenic LTi-deficient mice (84), many studies showed that key molecules, such as LT, CCL21, or CXCL13 chemokines, play also a major role in lymphoid neogenesis. Grabner *et al*. for example demonstrated in a mouse model of atherosclerosis that the activation through LTβR of medial smooth muscle cells (SMCs) in the abdominal aorta led to the expression of CCL19, CCL21, CXCL13, and CXCL16 chemokines, which in turn triggered the recruitment of lymphocytes to the adventitia and the development of TLS (85). In this study, authors suggest that the cellular source of LT would be CD11c^+^CD68^+^Ly6C^lo^ monocytes that accumulate in atherosclerotic plaques. Interestingly, Thaunat *et al*. showed in a rat model of chronic allograft rejection based on aorta transplantation that macrophages, together with CD4^+^ and CD8^+^ T cells, are one of the first immune cell subsets infiltrating the adventitia few days after engraftment, simultaneously with the acquisition by some endothelial cells of a HEV-like phenotype (37). The same group demonstrated that macrophages, in particular M1-polarized pro-inflammatory macrophages that express high levels of LTα and TNFα, can act as LTi cells in diseased arteries and confer a LTo phenotype to vascular SMCs, but that this mechanism is TNF receptor 1/2 (TNFR1/2) signaling-dependent and LTβR-independent (86). They explained the discrepancies observed between their study and others claiming that LTβR is crucial for the formation of TLS by the major role played by LTβR in inducing CXCL13 production, cytokine not indispensable to induce TLS in their model. Similarly, Moyron-Quiroz *et al*. were able to observe the formation of i-BALTs in mice upon influenza infection even in the absence of LTα, but not as well organized as the ones generated in wild-type mice, with B-cell areas devoid of FDCs, few intermixed T cells, and no expression of PNAd (21). Finally, in human NSCLC, we were able to detect the expression of a set of chemoattractants associated with T lymphocytes in TLS, including the previously cited chemokines CCL19, CCL21, and CXCL13 (53).

In accordance with the aforementioned role of DCs in lymphocyte homing to LNs, it was suggested that DCs could promote lymphocyte tumor infiltration through the regulation of tumor HEVs (87). Indeed, Martinet *et al*. showed that LTβ was specifically overexpressed in breast cancers characterized by a high density of HEVs, and that the major producers of LTβ in the tumor microenvironment were DCs. They notably showed that LTβ expression correlated with that of HEV-associated chemokines, and that DC-Lamp^+^ DC density correlated with HEV density, lymphocyte infiltration, and favorable clinical outcome (88). In mice, CD11c^+^ DCs were shown to play a major role in the maintenance of i-BALTs. Halle *et al*. for example demonstrated a massive infiltration of CD11c^+^ DCs followed by i-BALT neogenesis in the lung of modified vaccinia virus Ankara (MVA)-infected mice, and that the depletion of these cells after i-BALT formation resulted in strong i-BALT size reduction (89). Similarly, GeurtsvanKessel *et al*. showed in a mouse model of influenza virus infection that CD11c^hi^ DCs are essential for the maintenance of virus i-BALTs after virus clearance, as DC depletion resulted in reduced i-BALT numbers, associated with reduced B-cell density within the remaining clusters, and altered humoral immunity (49). This effect was associated with LTβ expression by DCs, as blocking LTβR signaling had the same effect as DC depletion. Inhibition of LTβR signaling was associated with reduced secretion of CXCL13, suggesting that DCs maintain i-BALTs by providing a continuous source of LTβ that is necessary for high CXCL13 production and retention of B cells. Interestingly, authors showed that repeated injections of DCs also led to the development of i-BALTs into naïve mice suggesting that DCs may have a direct action in lymphoid neogenesis. Finally, Marinkovic *et al*. showed in a CCL21-transgenic-LTi-deficient mouse model that the formation of thyroid-associated TLS started by the clustering of host tissue DCs with newly entered mature CD3^+^ CD4^+^ T cells, followed by the formation of PNAd^+^ HEVs (84).

It has been suggested that Th17 cells could also play a major role in TLS induction. Th17 cells and associated cytokines are elevated in a number of autoimmune and inflammatory diseases, in which their function is well documented [reviewed in Ref. (90)]. Their role in cancer however is more uncertain, due to their strong plasticity [reviewed in Ref. (91)]. The putative role of Th17 cells in TLS development notably comes from the observation that Th17 cells share many developmental and effector markers with LTi cells. It includes the nuclear hormone receptor retinoic acid-related orphan receptor γt (RORγt), which promotes the production of IL-17 and IL-22 by Th17 cells, LTi cells, and other RORγt^+^ innate lymphoid cells (ILCs); the chemokine receptor CCR6, which favors the recruitment of Th17 cells to the site of inflammation in response to its ligand CCL20; and the ligand-dependent transcription factor, aryl hydrocarbon receptor (AHR). Above all, both cell subsets express LTα1β2 on the cell membrane [reviewed in Ref. (92)]. The role of Th17 cells in TLS induction was particularly studied in mouse models of lung infection or autoimmunity. Rangel-Moreno *et al*. for example demonstrated in mice sensitized with LPS that i-BALT formation was dependent on IL-17 production by T cells, including Th17 cells and γδ T cells, while LTi cells were not necessary (93). Opposite results were obtained by Fleige et al., who showed in MVA-infected mice that i-BALT formation was independent of IL-17 (94). The same authors further deciphered this duality in another study, in which they suggested that the role played by IL-17 in i-BALT development depends on the type of inflammation-inducing pathogen (95). Actually, they showed that while mice infected with MVA developed in their lungs highly organized i-BALTs, with densely packed B-cell follicles characterized by CXCL12-producing stromal cells and a network of CXCL13^+^ FDCs, mice infected with *P. aeruginosa* developed i-BALTs with B-cell follicles that contained CXCL12-expressing stromal cells but lacked FDCs. Authors demonstrated that in the case of *P. aeruginosa* infection, IL-17 drives the differentiation of lung stroma toward CXCL12-expressing cells, which allows B-cell recruitment and follicle formation even in the absence of FDCs. Again, the major source of IL-17 was identified here as being γδ T cells within i-BALTs. Still in favor of IL-17 involvement in TLS induction, Peters *et al*. showed in a mouse model of multiple sclerosis [experimental autoimmune encephalomyelitis (EAE)] that the transfer of differentiated myelin oligodendrocyte glycoprotein (MOG)-specific Th17 cells was more capable of inducing ectopic lymphoid structures than other Th subtypes (96). Ectopic lymphoid structure development was notably associated here with the acquired expression by transferred Th17 cells of Tfh cell-associated molecules, such as CXCR5, ICOS, and Bcl6, and dependent on the expression of IL-17 by Th17 cells. Finally, in humans, Deteix *et al*. suggested that Th17 cells, and IL-17 and IL-21 secretion by these cells, promote lymphoid neogenesis within human renal grafts, and are associated with fast chronic rejection (97). They notably showed that while rejected renal grafts from slow progressor patients were characterized by the presence of IL-10-secreting regulatory T cells (Tregs), which positively correlated with graft survival, rejected renal grafts from fast progressor patients were characterized by the presence of IL-17- and IL-21-secreting Th17 cells, which negatively correlated with graft survival. In addition, the presence of Th17 cells correlated with the presence of AID^+^ GC B cells suggesting that Th17 cells promote intragraft neogenesis through the production of IL-21, which in turn stimulates intragraft B cells, with deleterious consequences on kidney transplant.

In accordance with the aforementioned study, it has been proposed that Tregs could impair TLS formation. Kocks *et al*. for example observed that CCR7-deficient mice spontaneously develop very organized BALTs under steady-state conditions (98). Because BALT occurrence was reverted by the adoptive transfer of CCR7-expressing wild-type Tregs, authors suggested that Tregs may play a role in controlling BALT formation. It is emphasized in this study that BALTs may not be *bona fide* TLS, as they arise in absence of any inflammatory signals. In parallel, Hindley *et al*. showed in carcinogen-induced tumor-bearing mice that Treg depletion allowed the development of HEVs within tumors, associated with higher expression of LTα, LTβ, CCL19, CCL21, and CXCL13, higher CD8^+^ T-cell infiltration, and lower tumor growth (99). These observations are in accordance with the study of Martinet *et al*., who showed a decreased FoxP3^+^/CD3^+^ T-cell ratio in HEV^hi^ human breast tumors (88).

Thus, if factors influencing TLS formation are still unclear, in particular in cancer, three critical events definitively appear to promote TLS formation: inflammatory cytokine production (such as LTα/β), lymphoid chemokine production, and HEV development (25). By their capacity to produce LT and pro-angiogenic factors, it appears that B cells and DCs may participate in the initiation and/or maintenance of TLS.

## Double Face of B Cells in Tumor Immunity

### Deleterious effects of B cells in anti-tumor immunity

For a long time, the deleterious or positive role of B cells in anti-tumor immune response was a matter of debate. Many studies reported a pro-tumoral role of B cells in cancer patients and tumor mice models. For example, the detection of specific antibodies in the serum or in the tumor of breast cancer patients was associated with poor prognosis (100). In advanced colorectal cancer, a reduction of the tumor size following B-cell depletion could be observed in half of the patients treated with Rituximab, a humanized monoclonal antibody directed against human CD20 (101). Several mechanisms have been proposed to explain this pro-tumoral effect, using inactivated genes, xenograft experiments, adoptive cell transfer, or systemic B-cell depletion in mice models. The participation and/or the maintenance of a chronic inflammation by B cells leading to the growth of the tumor have notably been suggested (102). Also, when bound to antigens into immune complexes, antibodies can activate the complement system, which can trigger inflammation in B-cell deficient (μMT) or B-cell depleted mice. Indeed, the secretion of mediators like anaphylatoxins (i.e., C3 and C5a components) can induce the recruitment of inflammatory cells which in turn may provide a rich pro-angiogenic and pro-tumoral environment (103). Depending on the context, immune complexes can also promote tumor growth *via* the degradation of the extracellular matrix and the promotion of angiogenesis in a granulocyte- and macrophage-dependent manner (104). B cells were shown to directly inhibit cytotoxic T-cell responses in colorectal, melanoma, and thymoma tumor models in μMT mice (105). In addition, B-cell-derived factors, like TGF-β and IL-10, can favor the differentiation and the recruitment of Tregs, further amplifying the immunosuppressive environment (106, 107). Lastly, it has been proposed that LT produced by B cells could favor tumor metastasis in an NF-κB-independent manner (74). This phenomenon was notably demonstrated in a mouse model of castration-resistant prostate cancer, in which tumor cell death induced by castration elicited an inflammatory response, associated with inflammatory chemokine production and recruitment of immune cells. In this model, newly recruited mature B2 cells induced the activation of prostate cancer cells *via* LT-LTβR signaling, followed by IKK-α nuclear translocation and STAT3 activation, ultimately enhancing androgen-independent tumor growth and spreading. However, some of these studies, like ones in μMT mice, have to be interpreted with caution, as mice used in these experiments have also profound defects in TCR repertoire usage and strong disruption of lymphoid tissue architecture with diminished FDC, DC, and NK cell numbers.

### Beneficial effects of B cells in anti-tumor immunity

Recent studies readdressed this question using more relevant animal models. For instance, DiLillo *et al*. demonstrated a higher growth of B16 melanoma and lung metastasis, associated with decreased numbers of both CD4^+^ and CD8^+^ effector T cells, in mice depleted in B cells using an anti-CD20 antibody as compared with wild-type mice, demonstrating the key role of B cells in the induction and maintenance of a protective anti-tumor cellular immunity (108). In accordance with a beneficial role for tumor-infiltrating B cells in some mouse models, numerous clinical studies reported a positive correlation between the densities of total B cells and the clinical outcome of cancer patients, such as in NSCLC (15), primary cutaneous melanoma (10), breast cancer (11, 12, 14), and ovarian cancer (18). Some studies included the organization of tumor-infiltrating B cells as a supplementary criteria to consider in addition to the cell density. Since then, markers of TLS B-cell follicles (follicular B cells, Tfh cells, B-cell chemoattractant CXCL13) were all associated with prolonged cancer patient survival (39, 43, 44). In chronic obstructive pulmonary disease (COPD), TLS B cells have been shown to secrete both CXCL13 and LT (109). Thus, it is tempting to speculate that intra-tumoral B cells may also play a beneficial role *via* the maintenance of cancer-associated TLS which are associated with long-term survival. The organization of B cells into TLS B-cell follicles may thus better reflect the initiation of a local anti-tumor B-cell-mediated immunity (18, 39, 110, 111). Actually, molecular analyses of TLS-derived GC B cells from patients suffering from RA, primary Sjögren syndrome, or myasthenia gravis showed evidence of oligoclonal B-cell proliferation and SHM of immunoglobulin variable genes (112–115). In RA, the functionality of TLS was further demonstrated by the presence of anti-citrullinated protein/peptide (ACPA) PCs surrounding ectopic lymphoid structures in the synovial tissue of patients (50). In metastatic melanoma patients, TLS B cells were antigen experienced as demonstrated by the presence of clonal amplification, somatic mutation, and isotype switching (40). We similarly detected in NSCLC patients all stages of B-cell differentiation within tumors, in accordance with the presence of AID^+^ GC B cells, differentiated memory B cells and plasmablasts within TLS, and PCs within tumor stroma (Figure 1) (39). The demonstration of a direct correlation between the percentage of PCs and the density of TLS follicular B cells in NSCLC tumors, together with the capacity of tumor PCs to secrete antibodies specific for tumor antigens (discussed below) were further evidence of TLS B-cell functionality.

In parallel of their capacity to produce antigen-specific antibodies, B cells can also act as powerful APCs for T-cell activation and memory T-cell development (116, 117). It is known that during antigen challenge, presentation of processed antigens by B cells to CD4^+^ T cells in the outer follicle is necessary to the full activation of B cells, and their differentiation into short-lived plasmablasts or their proliferation and organization into GC. Several ligand/receptor pairs are involved during the B-T-cell interaction and also determine the extent of primary expansion of CD4^+^ T cells, such as CD80-CD86/CD28, CD40/CD40L, and OX40L/OX40, expressed on the surface of B cells and T cells, respectively. Especially, interaction between CD40, that is constitutively expressed by B cells, and its ligand CD40L which is expressed by activated Th cells, is crucial for GC formation (47, 118). Besides of GC reaction, the contribution of B cells as APCs was demonstrated in many disorders, including autoimmunity and allograft rejection. In multiple sclerosis for instance, activated B cells were shown to stimulate activation and proliferation of myelin basic protein-specific autologous CD4^+^ T cells (119). Similarly, in non-obese diabetic (NOD) mice, Serreze et al. showed that B cells are necessary APCs for the initial priming of T-cell responses against the candidate pancreatic β cell autoantigen glutamic acid decarboxylase (GAD) (120). While NOD mice genetically deficient in B lymphocytes were resistant to T-cell-mediated autoimmune insulin-dependent diabetes mellitus, and failed to develop GAD-specific T-cell responses, repopulation of mice with B lymphocytes restored their ability to initiate anti-GAD T-cell immunity. This observation was confirmed by Bouaziz *et al*. who showed that B-cell depletion with an anti-CD20 antibody impaired CD4^+^ T-cell activation and clonal expansion in response to protein antigens and pathogen challenge, suggesting the requirement of both B cells and DCs for optimal antigen-specific CD4^+^ T-cell priming (121). This result is in accordance with our observation in NSCLC patients, in which high densities of both TLS mature DCs and TLS B cells correlated with longer survival (39). However, TLS mature DC^High^ patients do not match perfectly with TLS B-cell^High^ patients. The combination of both biomarkers was the best predictor for survival suggesting that both APCs may play a complementary role in the initiation of protective immune responses in NSCLC patients. The capacity of B cells to cross-present antigens to CD8^+^ T cells is also well established (122–124). We notably showed that 30-mer polypeptides derived from the cancer-testis antigen NY-ESO-1 were efficiently cross-presented by B cells and immunogenic, in that they induced the proliferation of nonamer-specific CD8^+^ T cells *in vitro* (122). Finally, B cells would have the capacity to reactivate T cells previously primed by DCs (125). According to Nielsen *et al*., B cells may similarly serve as APCs in cancer and thus promote anti-tumor T-cell responses (18). Actually, authors observed that tumor-infiltrating CD20^+^ B cells were often found in close proximity to CD8^+^ T cells, and that the presence of both CD20^+^ and CD8^+^ lymphocytes was associated with markedly prolonged survival of ovarian cancer patients compared with that of CD8^+^ T-cell infiltrate alone. In addition, they observed that tumor CD20^+^ B cells expressed several markers of antigen presentation, such as MHC Class I and II molecules, as well as the costimulatory molecules CD80 and CD86. In accordance with that study, a high expression of CD40, CD80, and HLA-DR was also reported by Yasuda *et al*. on tumor-infiltrating B cells in primary lung cancer (126). Finally, some tumor-infiltrating B-cell subsets may act as killer cells through the expression of granzyme B and TRAIL, induce a Th1 polarization *via* the secretion of IFNγ and to a lesser extend IL12p40, as well as present processed antigens to T cells through the expression of MHC class I, MHC class II, and costimulatory molecules, as demonstrated in hepatocellular carcinoma patients (127).

Lastly, the ligation of CD27 expressed by B cells to CD70 present on cytotoxic T lymphocytes (CTLs) was deemed critical to sustain B-cell activation and immunoglobulin synthesis in an antigen-independent manner, as well as to promote CTL survival and proliferation (128). In contrast to ovarian and hepatocellular carcinomas (18, 127), memory B cells infiltrating lung tumors are positive for CD27, which argues for an additional anti-tumor immune function of intra-tumoral B cells (39).

As for T-cell infiltrate, it is necessary to take into account the activation status and the cellular organization of intra-tumoral B cells within the tumor microenvironment. The presence of TLS may represent a privileged site where specific naïve B cells can undergo their final differentiation into effector B cells, i.e., memory B cells and PCs, and thus may play a pivotal role in cellular- and humoral-mediated anti-tumor immunity.

## Antibody Responses to Tumor Antigens in the Periphery and *In situ*

It has been clearly established that tumor-infiltrating B cells, and in particular cancer-associated TLS B cells, contribute to tumor prognosis and are able to produce antibodies locally. Thus, the important question to address is what these antibodies do, what their specificity is, and whether produced antibodies may contribute to the clinical outcome. In the 1980s, the knowledge that tumors can elicit spontaneous humoral responses spurred the development of autologous typing to identify novel molecular targets (129, 130). One of the most prolific methods to discover tumor-associated antigens was serological expression cloning (SEREX), which originally used serum of a cancer patient to screen for targets expressed in the autologous tumor after expression cloning (131). This unbiased method led to the discovery of a series of novel immunogenic targets, many of which were validated to be only eliciting antibody responses in the context of tumors (132). NY-ESO-1, discovered by SEREX, emerged as a model human tumor antigen, with restricted expression in normal tissues to germline cells, but widespread aberrant expression in various tumor types, and therefore named a cancer-testis antigen (133). Members of this family include MAGE, LAGE, XAGE, SSX, and other antigens, all of which are intracellular molecules, with cytoplasmic and/or nuclear expression. These antigens are controlled by epigenetic mechanisms and can be induced by hypomethylation or deacetylation of promoter sequences (134). It is thought that as tumors grow, some cells may be releasing antigens and initiating B-cell responses, and it is well established that detection of antibodies against these tumor antigens is associated with tumor antigen expression, even though there is no evidence that resulting antibodies have a direct functional role against tumors (135). Not much is known about what initiate immunogenicity, and whether it happens at the tumor site from local TLS, or whether more classic SLOs are responsible, after APCs carrying tumor extracts migrate there. A positive correlation has been described between size of LN B-cell areas and tumor-infiltrating B cells in head and neck cancer patients treated with a mixture of cytokines (136), suggesting that an interplay may be happening across SLOs and local tumor environment. To address the question of the structure initiating humoral responses, one would need a dynamic follow-up of antigen and uptake, and a better characterization of TLS versus LN versus circulating B cells for their specificity and chemokine receptor expression.

While B cells at the tumor site may recognize tumor antigens, it is important to consider their role and potential clinical benefit in a dynamic fashion as tumors evolve over time. Expression of most cancer-testis antigens tends to increase with stage and grade of disease, as shown for melanoma, lung, myeloma, and ovarian tumors among others, thereby highlighting more aggressive, poorly differentiated tumors with usually worse outcome (137), but that also become immunogenic as a result of new antigenicity. A study has found that NY-ESO-1 protein expression in primary breast carcinoma and metastases correlates with brisk plasmacytic B-cell infiltration (138). It is likely however that by the time, the tumor has reached a large enough size to generate B-cell response, the resulting antibodies are insufficient to affect the disease. Recently, tumor-infiltrating B cells were found increased in prostate cancer tissue with high intra-tumoral density of B cells related to higher stage and potential for recurrence or progression (139). In addition, B cells from ascites of ovarian cancer are able to produce antibodies to known tumor antigens, though the presence of B cells in ascites has been shown to increase with stage and to correlate with poor prognosis, but to decrease by chemotherapeutic treatment (140).

It is therefore important to view antibody responses as diverse repertoires with polyfunctionality, not all of it associated with better prognosis. Historically, circulating antibodies to tumor-specific antigens were seen as a sign that cellular immunity was skewed in favor of humoral rather than cellular responses, while CTLs were preferred for their direct anti-tumor capacity (103, 141). Mutated TP53 was one of the first tumor-associated antigens to be identified serologically (142), but circulating CTL responses against TP53 appear to be rare (143). Despite abundant literature emphasizing the split between B cell and Th2-mediated immunity versus cytotoxic CD8^+^ T cell and Th1 immunity, this dichotomy may not be so clear for all tumor-associated antigens. In particular, NY-ESO-1, one of the most spontaneously immunogenic antigens from the cancer-testis family, is an example for which presence of specific circulating antibodies typically implies concomitant presence of circulating effector-like CD4^+^ and CD8^+^ T cells, in an integrated fashion (144, 145). Therefore, antibodies have been proposed as a surrogate for T-cell immunity to NY-ESO-1. We showed that patients seropositive for NY-ESO-1 have NY-ESO-1-specific T cells accumulating at the tumor site in ovarian cancer patients, though their activity was blunted by expression of co-inhibitory molecules CTLA-4, PD-1, and LAG-3 (146). This may explain the lack of anti-tumor effect, and therapeutic strategies to reverse such responses will be discussed later. Nevertheless, these results suggest that seropositivity could be a surrogate for the presence of tumor-specific T cells infiltrating tumors.

Serum reactivities against tumor antigens have been described in many cancer types, such as anti-NY-ESO-1 antibodies in sarcoma (147) and primary breast cancer (148), anti-XAGE-1b antibodies in NSCLC (3), anti-NY-ESO-1, anti-LAGE-1, and anti-P53 antibodies in ovarian cancer (18, 149). Very recently, Ohue et al. showed that cancer patients with serum antibody-reactivity against the tumor antigen XAGE1 had prolonged overall survival as compared with seronegative patients (3). Similarly, antibodies to MUC1 appeared to confer better survival in various tumors, while p53 antibodies did the opposite (150). Circulating antibody responses to NY-ESO-1, SOX2, Ubiquilin, and others have been proposed as biomarkers for early detection of NSCLC (151, 152), though few have been validated to date (153). Presence of circulating autoantibodies to tumor antigens has also been well described in paraneoplastic syndrome, where specificities shared between tumors and the nervous system abruptly lead to autoimmune pathology, albeit with some anti-tumor benefit (154).

Some very recent studies have provided evidence that tumor-specific humoral responses can be generated *in situ*, within TLS. To characterize the specificity of local B cells at the tumor site, we performed *ex vivo* cultures of NSCLC-infiltrating B cells and showed that they were able to produce high levels of IgG and IgA directed against tumor antigens of the cancer-testis antigen family, such as LAGE-1, MAGE antigens, and NY-ESO-1, but also P53. The reactivity we observed was polyclonal, as a same patient could display reactivities against several different tumor antigens (39). The presence of IgA at the tumor site is intriguing, as it differs from the usual IgG detected in the periphery, and detailed repertoire analyses are still needed to distinguish potential differences locally and systemically. More evidence of antigen-specific local B cells was found in ductal breast carcinoma where infiltrating B cells from TLS-like structures were microdissected and showed polyclonality and high mutation rates, with some dominant clones shared between various clusters of B cells suggesting affinity maturation *in situ* and seeding from site to site (155). Finally, Maletzki *et al*. observed in colorectal cancer that tumor-infiltrating B cells produced IgG able to bind to the cell surface of several tumor cell lines (156).

Once again though, mere presence of antibodies may not necessarily be sufficient to predict outcome, as not all B-cell responses are of equal quality. In lung cancer, a recent report indicated increased circulating regulatory B cells [Bregs, reviewed in Ref. (157)] but decreased Tregs compared to healthy donors, highlighting a novel population of B cells to consider. It is likely that the context of the original antigen stimulation (viral versus self, chronically versus acutely inflamed) will drive the differentiation of the B-cell repertoire, and this opens up strategies for manipulation with immunotherapies.

## Toward TLS- and Antibody-Based Therapeutic Strategies in Cancer

It is assumed that the presence of TLS within tumors (except in renal cell carcinoma) is always associated with prolonged survival (27). Thus, a big challenge in the near future will be to develop new therapeutic tools, or to determine therapeutic combinations, able to favor the formation of such lymphoid structures in cancer patients.

Several studies have already reported the development of TLS as a consequence of antigen challenge during anti-tumor vaccination protocols. Lutz *et al*. for example observed the induction of TLS in pancreatic ductal adenocarcinoma (PDAC) after vaccination with GM-CSF-secreting pancreatic tumor vaccine (GVAX), a granulocyte-macrophage colony-stimulating factor (GM-CSF)-secreting, allogeneic PDAC vaccine (158). While PDAC is classically considered as a non-immunogenic neoplasm, tumors from patients vaccinated with GVAX presented organized and functional TLS as soon as 2 weeks following GVAX treatment. These TLS displayed all features of an ongoing immune response, including GC B-cell proliferation, T-cell activation, and Th1 transcription factor T-bet expression, associated with the induction of IFNγ-expressing effector T-cell responses. The number of TLS detected within PDAC tumors tended to increase when patients were treated with GVAX combined to the Treg-depleting chemotherapy cyclophosphamide, in accordance with studies showing that Tregs can suppress the development of TLS (88, 98, 99, 159). More interestingly, a downregulation of genes involved in the Treg pathway and a concomitant upregulation of genes involved in the Th17 pathway was detected in TLS of patients who survived longer and had enhanced immune responses. These results are in accordance with studies claiming that, as opposed to Tregs, Th17 cells and Th17 cytokines promote TLS development (92, 93, 96, 97). Similarly, Maldonado *et al*. observed the formation of TLS in patients with high-grade cervical intraepithelial neoplasias (CIN2/3) who received intramuscular therapeutic vaccination targeting HPV16 E6/E7 antigens (160). These TLS were associated with HEVs, immune cell activation and Th1 polarization signatures, and TCR clonality. In both studies, it seems that vaccination-induced antigen-specific T cells generated systemically were able to massively infiltrate the tumor microenvironment, and to initiate a local inflammatory reaction, further favoring the recruitment of immune cells and TLS neogenesis.

Besides vaccination protocols, which aim to increase humoral and cellular immune responses against particular tumor antigens, several studies demonstrated that some chemotherapeutic drugs have the capacity to enhance tumor immunogenicity and recognition by the immune system. Kroemer and Zitvogel groups notably have extensively documented how anthracyclines and oxaliplatin are able to trigger an immunogenic form of tumor cell death, and thus to promote anti-tumor immune responses, through three molecular mechanisms (161): first, the translocation of calreticulin on the tumor cell surface, which results in tumor engulfment by DCs; and secondly, the release by dying tumor cells of HMGB1 protein and ATP, two danger signals that bind TLR4 and P2X7 on DCs, respectively, and increase their ability to present tumor antigens and to activate anti-tumor CD8^+^ T-cell responses (162–164). Similarly, some DNA demethylating agents showed capacities to increase tumor immunogenicity, by favoring the expression of cancer-testis antigens in cancer cells (165). Ayyoub *et al*. for example demonstrated that DNA demethylation induced by 5-aza-2′-deoxycytidine (5-AZA-CdR) increased or induced mRNA expression of several cancer-testis antigens in sarcoma cell lines, including MAGE-A10, SSX, NY-ESO-1, and LAGE-1, resulting in increased tumor cell recognition by CTLs (147). Considering the potential role played by DCs and B cells in TLS induction and/or maintenance, we can speculate that chemotherapeutic drugs favoring DC and/or B-cell activation may also have a beneficial effect on the development of TLS within the tumor.

Other developed strategies aim to directly induce the formation of TLS within tumor tissue, by targeting at the tumor site signals or chemokines known as favoring immune cell recruitment and/or lymphoid neogenesis. Schrama *et al*. used the property of LTα to promote TLS development in a mouse model of melanoma (166). They developed an anti-disialoganglioside GD2 specific antibody-LTα fusion protein, and demonstrated that this fusion protein was able to favor subcutaneous tumor and established pulmonary metastasis eradication, and to prolong survival. Twenty-eight days after treatment, they could observe the presence of T cells and B cells organized in lymphoid-like patterns within tumors, characterized by the presence of MHC class II^+^ cells resembling DCs, the presence of CD62L^+^ cells in close contact with PNAd^+^ HEVs. Lymphoid neogenesis in the treated mice was associated with anti-tumor T-cell clonal expansion. Similarly, Liang *et al*. used the properties of the lymphoid chemokine CCL21 to favor the recruitment of immune cells at the tumor site (167). In a mouse model of hepatocellular carcinoma, they showed that the transduction of tumor cells with a recombinant adeno-associated virus (rAAV) expressing CCL21, or intra-tumoral injections of rAAV-CCL21, allowed extensive infiltration of the tumor site by CD11c^+^ DCs and activated CD3^+^ CD69^+^ T cells. Finally, Irvine *et al*. discussed the possibility of using tissue engineering, and matrix or scaffold-based approaches in order to create an implantable microenvironment that would support the recruitment of immune cell and/or favor lymphoid neogenesis through, for instance, the slow release of chemoattractants (168).

We can hypothesize that strategies aiming at increasing or re-activating exhausted T-cell responses, by targeting immunoregulators such as CTLA-4 or PD-1, may also have bystander effects on TLS development. Beyond immune checkpoint inhibitors, next generation immunomodulators may also involve costimulatory agonists that can target both T and B cells, including 4-1BB, CD27, HVEM, and CD40 (169). Cross-linking capacity of B cells was shown to be important for the therapeutic effect of immunomodulatory antibodies such as anti-CD40 (170). These novel strategies will need to be studied in detail for their role on local immune infiltration and organization, through the design of presurgical, neoadjuvant clinical studies. Another immunomodulatory approach, beside systemic administration of antibodies targeting immune pathways, may be to proceed with intra-tumoral injections of pathogen-associated molecular patterns (PAMPs) or cytokines, to change the inflammatory environment of the tumor. BTLA was described as an inhibitor of some B-cell functions that is upregulated in the context of immunotherapies including CpG administration, but could be actionable with blocking of its interaction with natural ligand HVEM (171). Intra-tumoral injection of IL-12 was also shown to activate B cells and lead to the polarization of immunoglobulin secretion to Th1-associated IgG1, along with IFNγ production by B cells (17).

The detection of a humoral anti-tumor immune response initiated within TLS and its characterization open the way to the development of new vaccine and antibody-based therapeutic strategies. As discussed above, reactivity against tumor antigens belonging to the cancer-testis antigen family is a feature shared by several cancer types, making these antigens as potential targets in future protocols of immunotherapy. Despite their intracellular expression, vaccines or monoclonal antibodies directed against some of these antigens have already been validated in preclinical models. Noguchi *et al*. for example demonstrated in mice the anti-tumor effect of an anti-NY-ESO-1 antibody when associated with chemotherapy (172). In this study, they observed not only the generation of an anti-NY-ESO-1 CD8^+^ T-cell immune response, but also the differentiation of other antigen-specific CD8^+^ T cells, thanks to antigen spreading and DC activation by anti-NY-ESO-1 immune complexes (173).

MAGE-A3 and NY-ESO-1 have entered into clinical trials for various cancers, and belong to the top 10 of the cancer antigens referenced in the “Prioritization of Cancer Antigens” project proposed by the National Cancer Institute (NCI) (174–177). Several studies have already reported the development of both humoral and cellular specific immune responses in NY-ESO-1-vaccinated cancer patients (144, 178). In one study, it was suggested that patients with the highest humoral responses following vaccination with NY-ESO-1 protein and CpG were more likely to have associated induction of CD8^+^ T cells (179).

Finally, as described above, NY-ESO-1 is known to elicit spontaneous immune responses though their clinical benefit is not clear, since the effector T cells at the tumor site may be dysfunctional due to immune checkpoint inhibition (146). We asked if some of the success of CTLA-4 blockade could be attributed to a change in immune responses against NY-ESO-1, since it is a frequent antigen in melanoma. We found that patients with pre-existing serological immunity to NY-ESO-1, coupled with detectable CD8^+^ T cells in the periphery, were nearly twice as likely to experience clinical benefit to ipilimumab compared to patients without NY-ESO-1 immunity (180). Circulating antibody titers, CD8^+^ T-cell repertoire, and polyfunctionality to NY-ESO-1 increased after treatment, but it is still unknown how ipilimumab affected local tumor B and T-cell responses. However, the clinical benefit suggests that having the tools to recognize NY-ESO-1 at baseline will make it more likely that immunomodulatory treatment will work.

## Concluding Remarks

Tertiary lymphoid structures in human tumors contribute to an environment conducive to the generation of B-cell diversity, with the help of T cells and DCs that leads to accumulation of tumor-specific immune responses inside or in proximity to the tumor. The presence of such lymphoid structures, including their B-cell component, underlines a population of patients that have already been primed to react against their tumors, albeit immunosurveillance has failed by the time the tumor is clinically detectable likely due to many counter-regulatory mechanisms. Nevertheless, TLS, along with T cells, mature DCs, and B cells, often predict eventual outcome in various diseases such as NSCLC, which suggests that having a primed tumor immune microenvironment, i.e., local immunocompetence, prior to treatment may facilitate various interventions and slow down progression. However, with progression also comes a series of counter-regulatory mechanisms that hamper the orchestrated anti-tumor immune responses, including the B-cell repertoire.

Many questions remain to be answered however, such as identifying whether immune responses are originally primed locally in TLS and become systemic, or conversely are generated in classic lymphoid organs and migrate to the disease site. Investigating differences in antigen cross-presentation, in antigen specificity locally and systemically, in quality and function between tumor B cells, LN B cells, and circulating B cells should help bring some answers.

## Conflict of Interest Statement

The authors declare that the research was conducted in the absence of any commercial or financial relationships that could be construed as a potential conflict of interest.

## Abbreviations

5-AZA-CdR, 5-aza-2′-deoxycytidine; ACPA, anti-citrullinated protein/peptide; ADCC, antibody-dependent cellular cytotoxicity; AHR, aryl hydrocarbon receptor; AID, activation-induced cytidine deaminase; APC, antigen-presenting cell; BALT, bronchus-associated lymphoid tissue; CDC, complement-dependent cytotoxicity; CSR, class switch recombination; CTL, cytotoxic T lymphocyte; DC, dendritic cell; EAE, experimental autoimmune encephalomyelitis; FcR, fragment constant receptor; FDC, follicular dendritic cell; FRC, fibroblastic reticular cell; GAD, glutamic acid decarboxylase; GC, germinal center; GD2, disialoganglioside 2; GM-CSF, granulocyte-macrophage colony-stimulating factor; GVAX, GM-CSF-secreting pancreatic tumor vaccine; HEV, high endothelial venule; i-BALT, induced BALT; ICAM-1, intercellular cell adhesion molecule 1; ILC, innate lymphoid cell; LN, lymph node; LTi, lymphoid tissue-inducer; LTo, lymphoid tissue-organizer; LTβ, lymphotoxin β; MALT, mucosal-associated lymphoid tissue; MHC, major histocompatibility complex; MOG, myelin oligodendrocyte glycoprotein; MVA, modified vaccinia virus Ankara; NALT, nasal-associated lymphoid tissue; NK, natural killer; NOD, non-obese diabetic; NSCLC, non-small cell lung cancer; PAMP, pathogen-associated molecular pattern; PC, plasma cell; PDAC, pancreatic ductal adenocarcinoma; pDC, plasmacytoid dendritic cell; PNAd, peripheral node addressin; RA, rheumatoid arthritis; rAAV, recombinant adeno-associated virus; SEREX, serological expression cloning; SHM, somatic hyper-mutation; SLO, secondary lymphoid organ; SMC, smooth muscle cell; Tfh, T follicular helper; Th, T helper; TLO, tertiary lymphoid organ; TLS, tertiary lymphoid structure; VCAM-1, vascular cell adhesion molecule 1; VEGF, vascular endothelial growth factor.
